# Supplementary material for: Compromised Blood-Brain Barrier Junctions Enhance Melanoma Cell Intercalation and Extravasation
Source: Cancers (Basel). 2023 Oct 20;15(20):5071. doi: 10.3390/cancers15205071 (PMC10605101; doi:10.3390/cancers15205071)
Supplement: Supplementary file 1 [file cancers-15-05071-s001.zip › Legend of Videos.pdf]

Supplementary Video S1. Intercalation of B78chOVA melanoma cells into TNF-α stimulated LifeAct-GFP$^+$ pMBMECs. Melanoma cells were accumulated on the pMBMECs under low flow (0.1 dyn/cm^2) for 4 min, followed by a 2-min pulse of 1.5 dyn/cm^2, which washed away all non-adherent melanoma cells. Then, the flow was stopped, and image acquisition was started to follow melanoma cell intercalation into the pMBMECs. Intercalation events were visualised by melanoma cell spreading visible in the phase contrast channel and the mCherry fluorescence channel and the displacement of the pMBMECs' LifeAct-GFP signal visible in the GFP fluorescence channel. Images were taken at 10-min intervals. The video was composed of four frames per second. The duration of the video is 4 s and corresponds to a real-time of 160 min. Left. Overlay of phase contrast, which is pseudo-coloured in yellow, highlighting the melanoma cells, and LifeAct-GFP signal of the pMBMECs, which is shown in white. Right. Only the LifeAct-GFP signal from the pMBMECs highlights the intercalation points. The timer is shown in the upper left corner. The scale bar is shown in the lower right corner. Images were acquired with an AxioObserver.Z1 microscope using the objective Plan-Neofluar 40×/0.6, the camera Axiocam 712 mono and the ZEN 3.4 blue software (Zeiss, Feldbach, Switzerland).

Supplementary Video S2. Intercalation of B78chOVA melanoma cells into TNF-α stimulated VE-CadGFP$^+$ pMBMECs. Melanoma cells were accumulated on the pMBMECs under low flow (0.1 dyn/cm^2) for 4 min, followed by a 2-min pulse of 1.5 dyn/cm^2, which washed away all non-adherent melanoma cells. Then, the flow was stopped, and image acquisition was started to follow melanoma cell intercalation into the pMBMECs, which became visible by a melanoma cell spreading and displacement of the VE-CadGFP signal from the pMBMECs (see Figure 2B,C). Images were taken at 6-min intervals. The video was composed of four frames per second. The duration of the video is 4.25 s and corresponds to a real time of 102 min. Left. Phase contrast overlay with the GFP channel to show the melanoma cells (phase contrast) and the VE-CadGFP signal of the pMBMECs. Second from left. Same as left, but with additional red mCherry fluorescence of the melanoma cells. Third from left. The VE-CadGFP signal only highlights the intercalation area. Right. The VE-CadGFP signal is overlaid with a red dotted line showing the contour of the intercalating melanoma cell. The timer is shown in the upper left corner. The scale bar is shown in the lower right corner. Images were acquired at an AxioObserver.Z1 microscope using the objective Plan-Neofluar 40×/0.6, the camera Axiocam 712 mono and the ZEN 3.4 blue software (Zeiss, Feldbach, Switzerland).

Supplementary Video S3. Intercalation of YUMM1.1-BrM4 melanoma cells into PECAM-1-wt or PECAM-1-ko LifeAct-GFP$^+$ pMBMECs. Melanoma cells were added to the pMBMECs under static conditions in a multi-well format. Imaging was at 5-min intervals and started 30 min after the melanoma cells were added to the pMBMECs. The video was composed with three frames per second. The duration of the video is 7.7 s and corresponds to a real time of 115 min. Upper row, LifeAct-GFP signal only to visualise the melanoma cell intercalation events by displacement of the GFP signal (white). The bottom row overlay of the LifeAct-GFP signal of the pMBMECs and the brightfield channel adjusted to highlight the melanoma cell contour. Left, PECAM-1-wt LifeAct-GFP$^+$ pMBMECs. Right, PECAM-1-ko LifeAct-GFP$^+$. The timer and sample description are shown on the top. The scale bar is shown in the lower right corner. Images were acquired at an INCell Analyzer 2000 using the built-in CCD camera, the 20× objective and the INCell Investigator v1.5 software (GE HealthCare, Chicago, IL, USA).

Supplementary Videos S4 and S5. Exemplary high-resolution imaging of one extravascular melanoma cell and one intravascular melanoma cell. Video S4 shows an extravascular melanoma cell detected in a brain slice of a PECAM-1-ko LifeAct-GFP$^+$ C57BL/6J mouse. Video S5 shows an intravascular melanoma cell detected in a brain slice of a PECAM-1-wt LifeAct-GFP$^+$ C57BL/6J mouse. Imaging was done with a Nikon A1 confocal microscope using the 60×/0.95 objective and the Nikon NIS-Elements software in 41 z-steps over 40 μm z-distance. The 3D animation was produced with NIS-Elements software using the shaded α blending method.
