# Supplementary material for: Compromised Blood-Brain Barrier Junctions Enhance Melanoma Cell Intercalation and Extravasation
Source: Cancers (Basel). 2023 Oct 20;15(20):5071. doi: 10.3390/cancers15205071 (PMC10605101; doi:10.3390/cancers15205071)
Supplement: Supplementary file 1 [file cancers-15-05071-s001.zip › Saltarin_et_al_Supplementary figures_Revision02.pdf]

Supplementary data to

Compromised blood-brain barrier junctions enhance melanoma cell intercalation and extravasation

Supplementary Figure S1

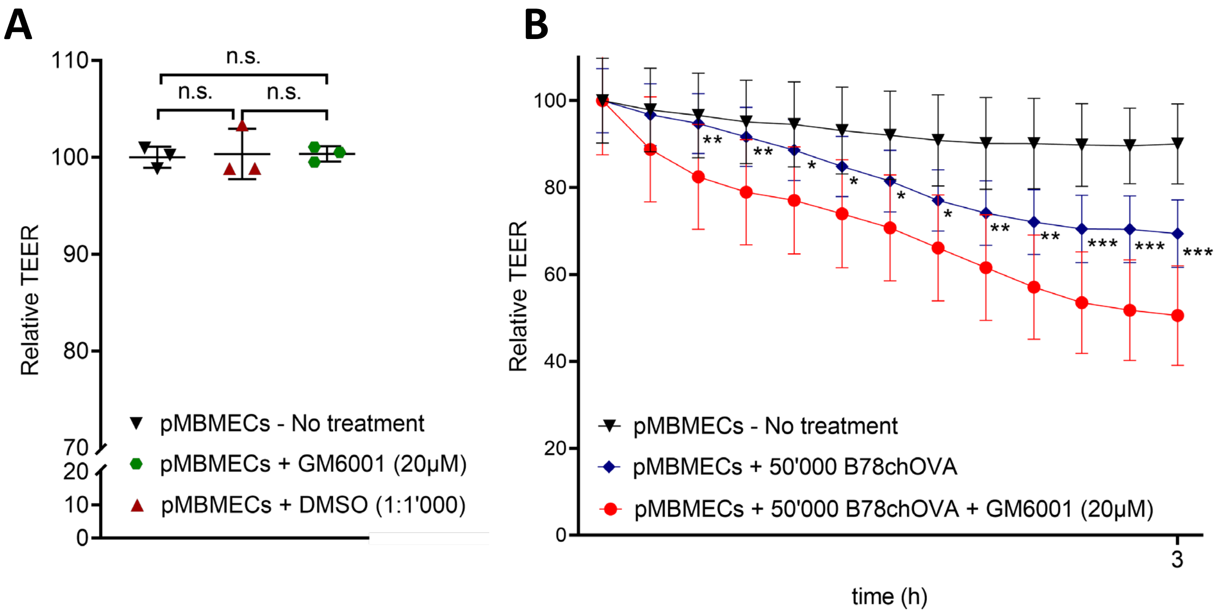

Supplementary Figure S1. Rescue of melanoma cell-induced barrier disruption of pMBMECs by protease inhibition. A. Relative TEER values of control pMBMEC samples at 3 hours after start of treatment: pMBMECs (▼ 2 experiments, each in triplicate), pMBMECs with DMSO (▲ 1 experiment in triplicate), pMBMECs with GM6001 (● 1 experiment in triplicate). Values are expressed relative to first pMBMECs, no treatment, whose mean was set to 100 %. Absolute TEER values and the calculation of data are shown in supplementary table 1. B. The TEER of pMBMECs (▼ 2 experiments, each in triplicate), pMBMECs with B78chOVA melanoma cells (♦ 1 experiment with 9 samples) or pMBMECs with B78chOVA melanoma cells plus GM6001 (● 1 experiment with 9 samples) measured in 15-minute intervals. Measurement started after start of treatment and was over 3 hours. The statistical significance of the differences in TEER values was calculated between pMBMECs +B78chOVA and pMBMECs +B78chOVA +GM6001 using the unpaired 2-sample t-tests for each time point. Values are expressed relative to the first time point whose mean was set to 100 %. Initial absolute values and the calculation of data are shown in supplementary table 2. A, B. Error bars show standard deviation.

Supplementary Figure S2

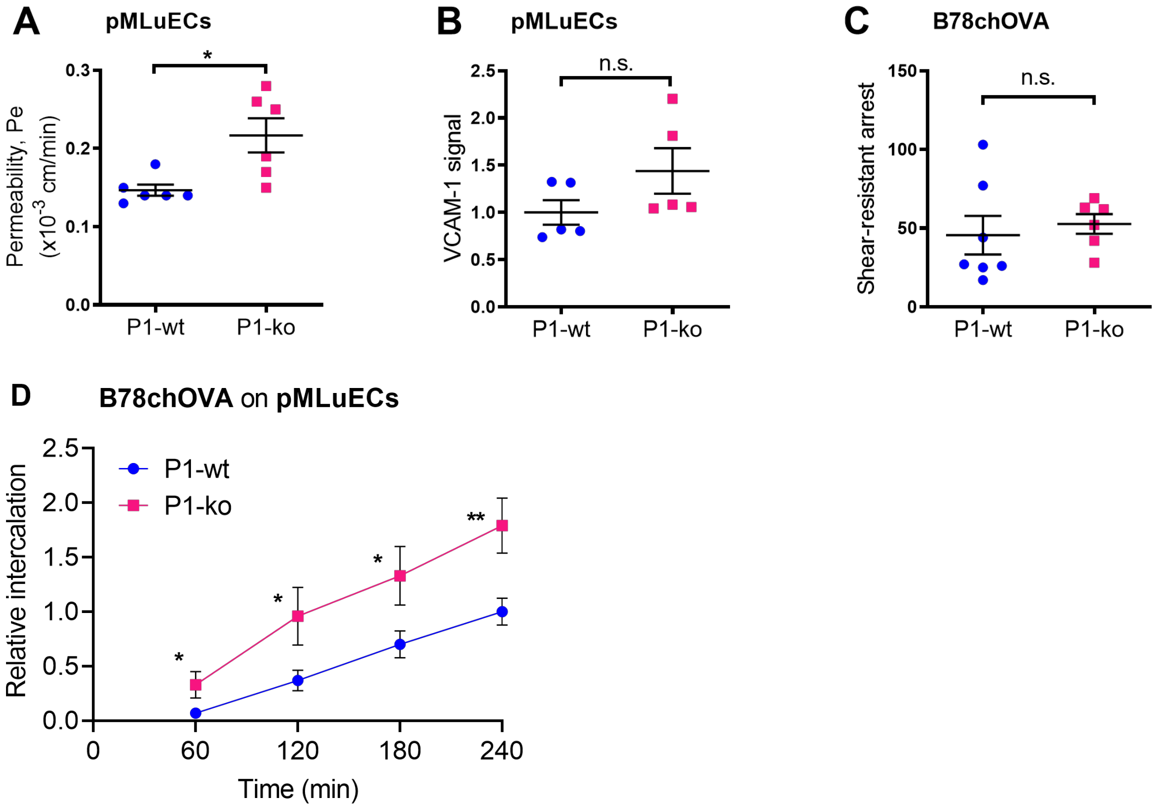

<u>*Supplementary Figure S2.*</u> PECAM-1 deficient pMLuECs show compromised endothelial barrier properties and increased B78chOVA melanoma cell intercalation but comparable VCAM-1 expression and similar melanoma cell adhesion. **A** Quantification of permeability to 10 kDa dextran of PECAM-1 (P1)-wt and P1-ko pMLuECs, stimulated with TNF-α (n = 2, each experiment in triplicates). **B** Quantification of VCAM-1 expression on TNF-α stimulated P1-wt or P1-ko pMLuECs, assessed by on-cell western. VCAM-1 fluorescence intensity of P1-wt pMLuECs was set to 1.0. (n = 5). **C** Quantification of B78chOVA melanoma cell adhesion to TNF-α stimulated P1-wt and P1-ko pMLuECs. Adhered melanoma cells were counted per field of view (6.1 x 10^5 µm) after a 30 – 60 second pulse of flow to remove non-adherent melanoma cells (n = 2). **D** Quantification of B78chOVA melanoma cell intercalation into TNFα-stimulated P1-wt and P1-ko pMLuECs. Intercalating B78chOVA melanoma cells per FOV (6.1 x10^5 µm^2) were counted 60, 120, 180 and 240 minutes after start of the experiment (n = 3, each experiment at least in duplicate). Experiments were performed in the 96-well static setup. P-values refer to the paired time points.

<u>*Methods to Supplementary Figure S2.*</u> pMLuECs were isolated using the immunomagnetic selection method as described before [1]. Instead of anti-PECAM-1, the anti-ICAM-2 antibody was used. Repurification was done twice with 2 weeks in between each time. The cells were split in 1 to 3 ratios every 10 days. All experiments were performed with pMLuECs of passage between 6 and 11. Melanoma cell intercalation was as described in the main manuscript with the exception that B78chOVA intercalation was assessed by visual inspection of the co-cultures in phase contrast mode for the image-time series acquired with an AxioObserver Z1 microscope with a Plan-Neofluar, 10x/0.3 objective (Carl Zeiss). The mCherry fluorescence of the melanoma cells was used to confirm melanoma cell identity.

1. Moore, T.L., Hauser, D., Gruber, T., Rothen-Rutishauser, B., Lattuada, M., Petri-Fink, A., and Lyck, R. "Cellular Shuttles: Monocytes/Macrophages Exhibit Transendothelial Transport of Nanoparticles under Physiological Flow". *ACS Appl Mater Interfaces* 9, (2017): 18501-18511.

Supplementary Figure S3

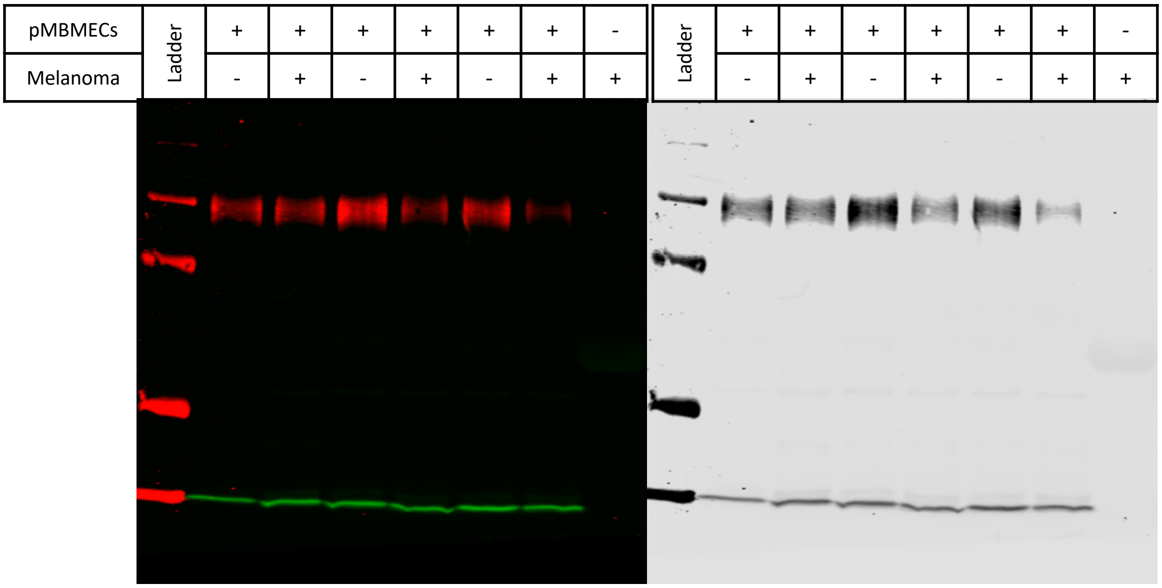

221214, Blot 1

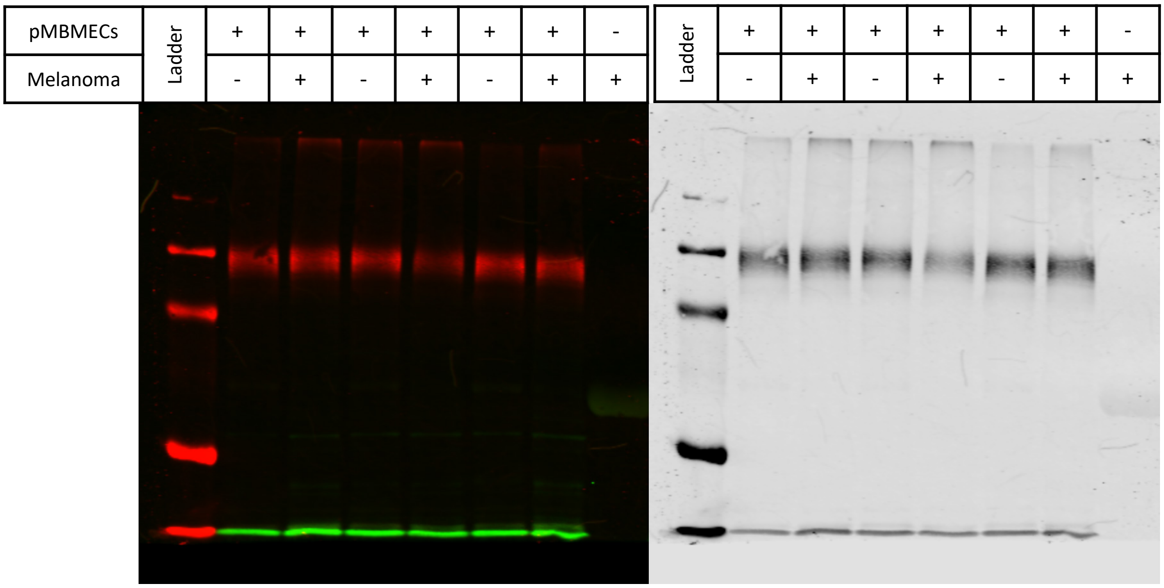

221214, Blot 2

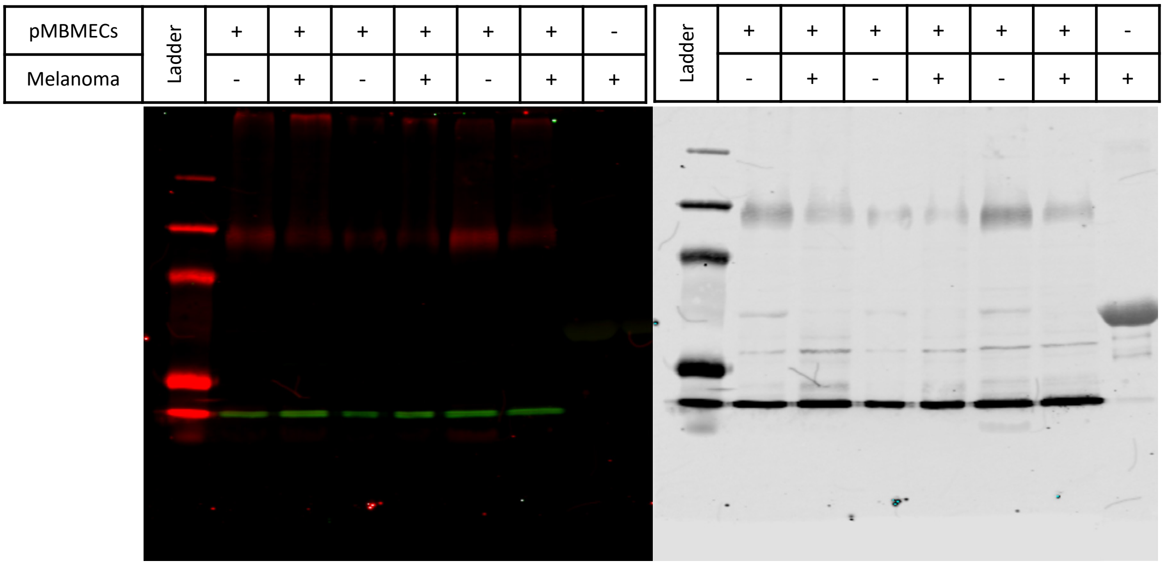

221214, Blot 3

Supplementary Figure S3, continued

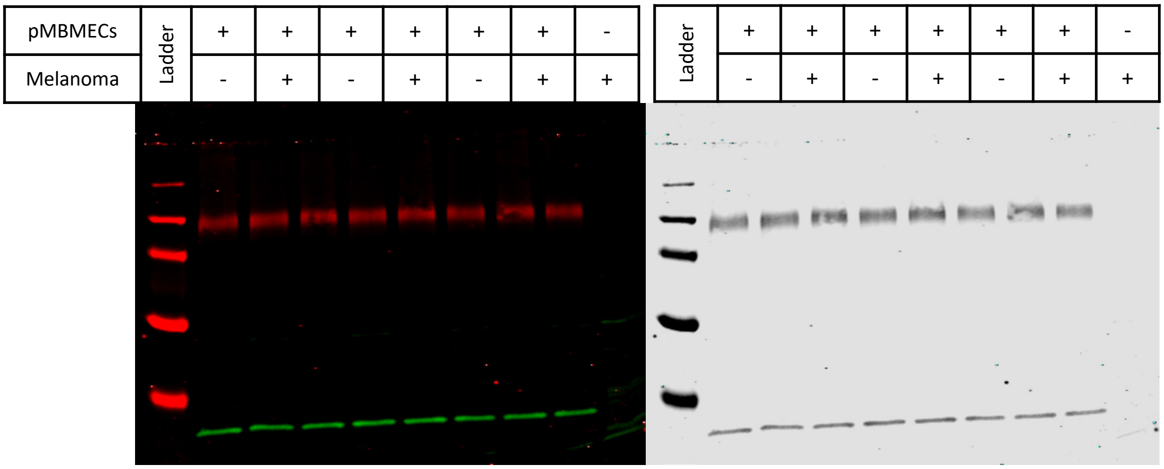

230207, Blot 1

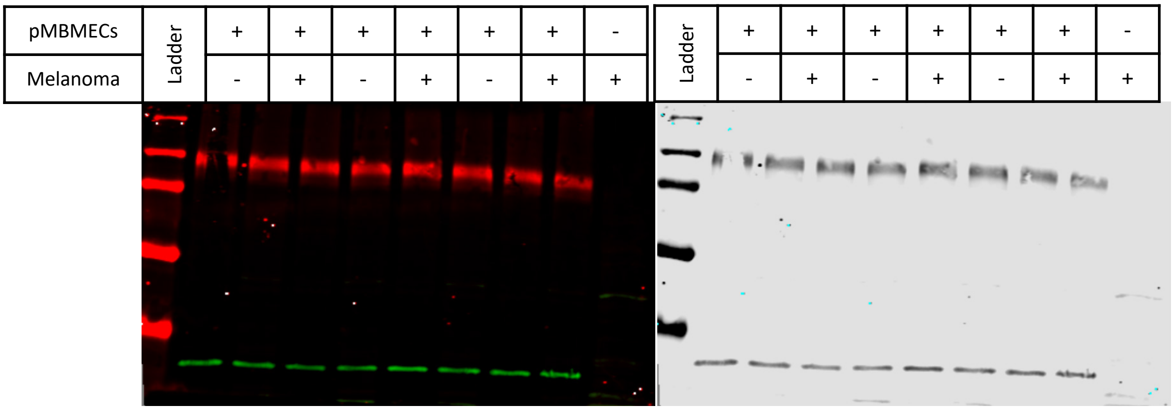

230207, Blot 2

Supplementary Figure S3. Original Western blots from 5 individual experiments to quantify PECAM-1 signal intensity from pMBMECs after incubation with melanoma cells, shown in Figure 8C. The left panels show the overlay of 2 channels in different colors: green, anti-GFP signal at 700 nm. Red, anti-PECAM-1 signal at 800 nm. The prestained molecular weight standard is also detected in the red channel recorded at 800 nm. The right panels show the grayscale image transformation from the colored overlay. The labels at the top of the blots describe the nature of each sample. Amounts of either 10 µg or 7.5 µg of protein were loaded per sample.

Supplementary Figure S4

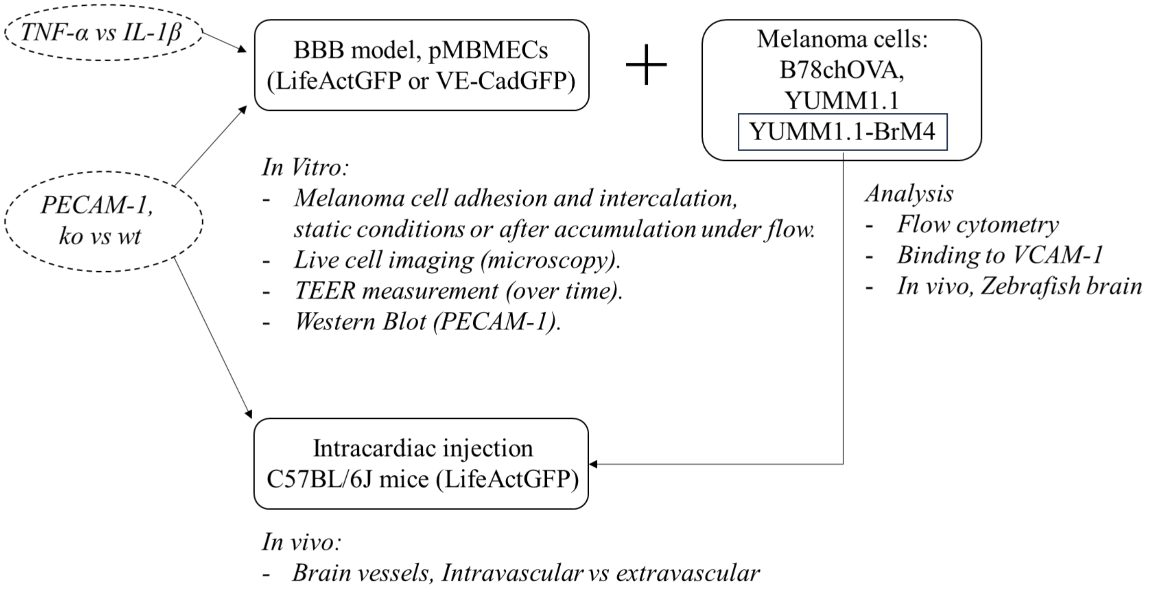

Supplementary Figure S4. Flow chart to illustrate the methodology. The BBB was modeled using pMBMECs isolated from LifeAct-GFP to analyze the number of melanoma cell intercalation events or from VE-CadGFP transgenic mice to analyze the melanoma intercalation pathway. To analyze the number of melanoma cell intercalation events into barrier-compromised pMBMECs, 2 models were used (dotted circles): (1) pMBMECs were stimulated with TNF-α or IL-1β. (2) pMBMECs were isolated from PECAM-1-wt or ko mice. B78chOVA, YUMM1.1 and YUMM1.1-BrM4 were used as melanoma cells. YUMM1.1-BrM4 melanoma cells were analyzed by flow cytometry, binding assays and in the zebrafish in vivo. Adhesion and intercalation of melanoma cells was studied under static conditions and after accumulation on the pMBMECs under physiological flow using live cell imaging. TEER was used to measure the barrier properties of the pMBMECs, and Western blot was used to test the PECAM-1 level of the pMBMECs. In vivo, the extravasation of YUMM1.1-BrM4 melanoma cells through cerebral vessels after intracardiac injection into syngeneic C57BL/6J LifeAct-GFP + PECAM-1-ko or PECAM-1-wt mice was tested.
