# Supplementary material for: Compromised Blood-Brain Barrier Junctions Enhance Melanoma Cell Intercalation and Extravasation
Source: Cancers (Basel). 2023 Oct 20;15(20):5071. doi: 10.3390/cancers15205071 (PMC10605101; doi:10.3390/cancers15205071)
Supplement: Supplementary file 1 [file cancers-15-05071-s001.zip › Saltarin_et_al_Supplementary tables_Revision02.pdf]

Supplementary tables to

Compromised blood-brain barrier junctions enhance melanoma cell intercalation and extravasation

Supplementary Table S1, calculation of data shown in Supplementary Figure S1A

A

	1st value after start of treatment					3 hours after start of treatment				
	ohm/cm2					ohm/cm2				
Sample	Value 1	Value 2	Value 3	mean	STDEV	Value 1	Value 2	Value 3	mean	STDEV
Empty filter	5,54	6,31	7,27	**6,4**	0,87	5,47	6,27	7,16	**6,3**	0,85
pMBMECs, no treatment	26,74	24,1	27,7	**26,2**	1,86	25,07	22,87	26,38	**24,8**	1,77
pMBMECs +GM6001 (20 µM)	22,16	23,24	22,63	**22,7**	0,54	20,85	21,85	21,97	**21,6**	0,61
pMBMECs +DMSO (1:1000)	19,57	22,46	21,17	**21,1**	1,45	18,67	21,46	20,03	**20,1**	1,40

B

	1st value after start of treatment *)					3 hours after start of treatment *)				
	ohm/cm2					ohm/cm2				
Sample	Value 1	Value 2	Value 3	mean	STDEV	Value 1	Value 2	Value 3	mean	STDEV
pMBMECs, no treatment	20,4	17,7	21,3	**19,8**	1,86	18,8	16,6	20,1	**18,5**	1,77
pMBMECs +GM6001 (20 µM)	15,8	16,9	16,3	**16,3**	0,54	14,6	15,6	15,7	**15,3**	0,61
pMBMECs +DMSO (1:1000)	13,2	16,1	14,8	**14,7**	1,45	12,4	15,2	13,7	**13,8**	1,40

*) mean of empty filter values subtracted

C

	1st value after start of treatment					3 hours after start of treatment				
	set to 100 %					relative to 1st value				
Sample	Value 1	Value 2	Value 3			Value 1	Value 2	Value 3	mean	STDEV
pMBMECs, no treatment	100	100	100			92,16	93,47	94,15	93,3	1,01
pMBMECs +GM6001 (20 µM)	100	100	100			92,17	92,19	96,39	93,6	2,43
pMBMECs +DMSO (1:1000)	100	100	100			93,74	94,24	92,79	93,6	0,74

	3 hours after start of treatment				
	**data shwon in Fig 3A**				
	Value 1	Value 2	Value 3	mean	STDEV
**)	98,82	100,23	100,96	**100,0**	1,09
***)	98,82	98,85	103,35	**100,3**	2,61
***)	100,51	101,05	99,49	**100,3**	0,79

**) mean of pMBMECs, no treatment, 3 hours after start of treatment set to 100 %.

***) TEER values are expressed relative to the mean of pMBMECs, no treatment, after 3 hours set to 100 %.

Supplementary Table S1. **A** lists the initial absolute TEER values. **B** lists the absolute TEER values subtracted by mean TEER values of the empty filters. **C** shows the calculation of the data shown in Supplementary Figure 1A.

Supplementary Table S2, calculation of data shown in Supplementary Figure S1B.

A

time (h)	Empty filter (Experiment A) 1	2	3	mean	STDEV	Empty filter (Experiment B) 1	2	3	mean	STDEV
	ohm/cm^2					ohm/cm^2				
start (0)	7,25	8,73	8,55	**8,18**	0,81	5,54	6,31	7,27	**6,37**	0,87
0.25	7,39	8,79	8,61	**8,26**	0,76	5,55	6,33	7,26	**6,38**	0,86
0.50	7,49	8,77	8,61	**8,29**	0,70	5,53	6,33	7,25	**6,37**	0,86
0.75	7,66	8,80	8,69	**8,38**	0,63	5,56	6,36	7,28	**6,40**	0,86
1.00	7,77	8,77	8,70	**8,41**	0,56	5,48	6,30	7,20	**6,33**	0,86
1.25	7,95	8,76	8,79	**8,50**	0,48	5,58	6,38	7,31	**6,42**	0,87
1.50	7,98	8,77	8,88	**8,54**	0,49	5,54	6,38	7,29	**6,40**	0,88
1.75	8,12	8,88	9,09	**8,70**	0,51	5,51	6,33	7,24	**6,36**	0,87
2.00	8,24	8,94	9,22	**8,80**	0,50	5,55	6,35	7,26	**6,39**	0,86
2.25	8,32	8,99	9,34	**8,88**	0,52	5,48	6,32	7,21	**6,34**	0,87
2.50	8,42	9,11	9,43	**8,99**	0,52	5,47	6,28	7,20	**6,32**	0,87
2.75	8,44	9,08	9,44	**8,99**	0,51	5,51	6,31	7,21	**6,34**	0,85
3.00	8,44	9,15	9,48	**9,02**	0,53	5,47	6,27	7,16	**6,30**	0,85

time (h)	pMBMECs, no treatment (Exp A) 1	2	3	mean	STDEV	pMBMECs, no treatment (Exp B) 1	2	3	mean	STDEV
	ohm/cm^2					ohm/cm^2				
start (0)	18,04	20,61	20,33	**19,66**	1,41	26,74	24,10	27,70	**26,18**	1,86
0.25	17,85	20,39	20,12	**19,45**	1,40	26,18	23,89	27,43	**25,83**	1,80
0.50	17,69	20,31	19,95	**19,32**	1,42	25,86	23,71	27,29	**25,62**	1,80
0.75	17,54	20,06	19,70	**19,10**	1,36	25,78	23,75	27,27	**25,60**	1,77
1.00	17,43	19,89	19,52	**18,95**	1,33	25,82	23,73	27,31	**25,62**	1,80
1.25	17,28	19,71	19,30	**18,76**	1,30	25,86	23,74	27,27	**25,62**	1,78
1.50	17,20	19,52	19,05	**18,59**	1,23	25,80	23,62	27,20	**25,54**	1,80
1.75	17,15	19,45	18,96	**18,52**	1,21	25,69	23,51	27,02	**25,41**	1,77
2.00	17,18	19,44	18,97	**18,53**	1,19	25,61	23,38	26,97	**25,32**	1,81
2.25	17,29	19,52	19,13	**18,65**	1,19	25,42	23,27	26,91	**25,20**	1,83
2.50	17,45	19,65	19,34	**18,81**	1,19	25,33	23,19	26,31	**24,94**	1,60
2.75	17,57	19,74	19,47	**18,93**	1,18	25,23	23,05	25,83	**24,70**	1,46
3.00	17,68	19,79	19,50	**18,99**	1,14	25,07	22,87	26,38	**24,77**	1,77

time (h)	pMBMECs +B78chOVA (Exp A) 1	2	3	4	5	6	7	8	9	mean	STDEV
	ohm/cm2										
start (0)	23,63	22,55	21,24	21,07	21,28	20,55	20,53	20,54	17,79	**21,02**	1,60
0.25	22,34	21,12	19,86	19,49	19,92	19,22	19,23	19,20	16,62	**19,67**	1,55
0.50	21,57	20,33	19,09	18,65	19,06	18,43	18,48	18,47	15,84	**18,88**	1,55
0.75	21,25	19,94	18,81	18,25	18,63	18,06	18,15	18,14	15,47	**18,52**	1,56
1.00	21,06	19,74	18,71	17,91	18,43	17,87	17,93	17,89	15,22	**18,31**	1,58
1.25	20,71	19,49	18,49	17,51	18,12	17,68	17,52	17,65	14,84	**18,00**	1,60
1.50	20,28	19,06	18,03	17,06	17,81	17,29	17,18	17,41	14,53	**17,63**	1,56
1.75	19,84	18,67	17,53	16,51	17,30	16,94	16,79	17,00	14,11	**17,19**	1,56
2.00	19,51	18,11	16,91	16,07	16,76	16,46	16,35	16,51	13,70	**16,71**	1,56
2.25	19,03	17,62	16,40	15,51	16,22	15,98	15,94	15,91	13,31	**16,21**	1,54
2.50	18,64	17,15	16,05	15,15	15,92	15,59	15,65	15,55	13,04	**15,86**	1,50
2.75	18,36	16,90	15,88	14,90	15,71	15,37	15,45	15,35	12,82	**15,64**	1,49
3.00	18,18	16,73	15,87	14,68	15,59	15,28	15,40	15,21	12,72	**15,52**	1,47

time (h)	pMBMECs +B78chOVA +GM6001 (20µM) (Exp A) 1	2	3	4	5	6	7	8	9	mean	STDEV
	ohm/cm2										
start (0)	19,80	20,53	19,46	19,85	19,19	20,81	18,42	20,20	18,43	**19,63**	0,85
0.25	19,61	20,22	19,19	19,49	18,94	20,50	18,20	19,85	18,15	**19,35**	0,82
0.50	19,42	19,92	19,05	19,25	18,71	20,28	18,04	19,64	17,99	**19,14**	0,79
0.75	19,15	19,62	18,80	18,97	18,47	20,05	17,82	19,36	17,71	**18,88**	0,78
1.00	18,85	19,34	18,48	18,65	18,18	19,73	17,47	19,06	17,32	**18,56**	0,81
1.25	18,51	19,02	18,12	18,34	17,83	19,33	17,18	18,72	16,95	**18,22**	0,80
1.50	18,18	18,72	17,77	18,00	17,50	19,00	16,79	18,38	16,60	**17,88**	0,81
1.75	17,85	18,38	17,33	17,67	17,15	18,59	16,46	18,05	16,23	**17,52**	0,81
2.00	17,71	18,22	17,06	17,43	16,97	18,40	16,16	17,76	15,95	**17,30**	0,85
2.25	17,64	18,06	16,82	17,30	16,84	18,22	16,01	17,59	15,76	**17,14**	0,86
2.50	17,58	18,05	16,67	17,24	16,79	18,14	15,91	17,57	15,63	**17,06**	0,89
2.75	17,63	18,00	16,62	17,25	16,80	18,07	15,87	17,57	15,65	**17,05**	0,88
3.00	17,59	17,92	16,57	17,20	16,78	17,97	15,69	17,45	15,58	**16,97**	0,89

B

time (h)	pMBMECs, no treatment (Exp A) 1	2	3	mean	STDEV	pMBMECs, no treatment (Exp B) 1	2	3	mean	STDEV
	ohm/cm^2					ohm/cm^2				
start (0)	9,86	12,43	12,15	**11,48**	1,41	20,37	17,73	21,33	**19,81**	1,86
0.25	9,59	12,13	11,86	**11,19**	1,40	19,80	17,51	21,05	**19,45**	1,80
0.50	9,40	12,02	11,66	**11,03**	1,42	19,49	17,34	20,92	**19,25**	1,80
0.75	9,16	11,68	11,32	**10,72**	1,36	19,38	17,35	20,87	**19,20**	1,77
1.00	9,02	11,48	11,11	**10,53**	1,33	19,49	17,40	20,98	**19,29**	1,80
1.25	8,78	11,21	10,80	**10,26**	1,30	19,44	17,32	20,85	**19,20**	1,78
1.50	8,66	10,98	10,51	**10,05**	1,23	19,40	17,22	20,80	**19,14**	1,80
1.75	8,45	10,75	10,26	**9,82**	1,21	19,33	17,15	20,66	**19,05**	1,77
2.00	8,38	10,64	10,17	**9,73**	1,19	19,22	16,99	20,58	**18,93**	1,81
2.25	8,41	10,64	10,25	**9,76**	1,19	19,08	16,93	20,57	**18,86**	1,83
2.50	8,46	10,66	10,35	**9,83**	1,19	19,01	16,87	19,99	**18,63**	1,60
2.75	8,58	10,75	10,48	**9,94**	1,18	18,89	16,71	19,49	**18,36**	1,46
3.00	8,66	10,77	10,48	**9,97**	1,14	18,77	16,57	20,08	**18,47**	1,77

time (h)	**pMBMECs +B78chOVA (Exp A)** 1	2	3	4	5	6	7	8	9	mean	STDEV
	ohm/cm2										
start (0)	**15,45**	**14,37**	**13,06**	**12,89**	**13,10**	**12,37**	**12,35**	**12,36**	**9,61**	**12,84**	**1,60**
0.25	**14,08**	**12,86**	**11,60**	**11,23**	**11,66**	**10,96**	**10,97**	**10,94**	**8,36**	**11,40**	**1,55**
0.50	**13,28**	**12,04**	**10,80**	**10,36**	**10,77**	**10,14**	**10,19**	**10,18**	**7,55**	**10,59**	**1,55**
0.75	**12,87**	**11,56**	**10,43**	**9,87**	**10,25**	**9,68**	**9,77**	**9,76**	**7,09**	**10,14**	**1,56**
1.00	**12,65**	**11,33**	**10,30**	**9,50**	**10,02**	**9,46**	**9,52**	**9,48**	**6,81**	**9,89**	**1,58**
1.25	**12,21**	**10,99**	**9,99**	**9,01**	**9,62**	**9,18**	**9,02**	**9,15**	**6,34**	**9,50**	**1,60**
1.50	**11,74**	**10,52**	**9,49**	**8,52**	**9,27**	**8,75**	**8,64**	**8,87**	**5,99**	**9,08**	**1,56**
1.75	**11,14**	**9,97**	**8,83**	**7,81**	**8,60**	**8,24**	**8,09**	**8,30**	**5,41**	**8,49**	**1,56**
2.00	**10,71**	**9,31**	**8,11**	**7,27**	**7,96**	**7,66**	**7,55**	**7,71**	**4,90**	**7,91**	**1,56**
2.25	**10,15**	**8,74**	**7,52**	**6,63**	**7,34**	**7,10**	**7,06**	**7,03**	**4,43**	**7,33**	**1,54**
2.50	**9,65**	**8,16**	**7,06**	**6,16**	**6,93**	**6,60**	**6,66**	**6,56**	**4,05**	**6,87**	**1,50**
2.75	**9,37**	**7,91**	**6,89**	**5,91**	**6,72**	**6,38**	**6,46**	**6,36**	**3,83**	**6,65**	**1,49**
3.00	**9,16**	**7,71**	**6,85**	**5,66**	**6,57**	**6,26**	**6,38**	**6,19**	**3,70**	**6,49**	**1,47**

time (h)	pMBMECs +B78chOVA +GM6001 (20µM) (Exp A) 1	2	3	4	5	6	7	8	9	mean	STDEV
	ohm/cm2										
start (0)	11,62	12,35	11,28	11,67	11,01	12,63	10,24	12,02	10,25	**11,46**	0,85
0.25	11,35	11,96	10,93	11,23	10,68	12,24	9,94	11,59	9,89	**11,09**	0,82
0.50	11,13	11,63	10,76	10,96	10,42	11,99	9,75	11,35	9,70	**10,85**	0,79
0.75	10,77	11,24	10,42	10,59	10,09	11,67	9,44	10,98	9,33	**10,50**	0,78
1.00	10,44	10,93	10,07	10,24	9,77	11,32	9,06	10,65	8,91	**10,15**	0,81
1.25	10,01	10,52	9,62	9,84	9,33	10,83	8,68	10,22	8,45	**9,72**	0,80
1.50	9,64	10,18	9,23	9,46	8,96	10,46	8,25	9,84	8,06	**9,34**	0,81
1.75	9,15	9,68	8,63	8,97	8,45	9,89	7,76	9,35	7,53	**8,83**	0,81
2.00	8,91	9,42	8,26	8,63	8,17	9,60	7,36	8,96	7,15	**8,50**	0,85
2.25	8,76	9,18	7,94	8,42	7,96	9,34	7,13	8,71	6,88	**8,25**	0,86
2.50	8,59	9,06	7,68	8,25	7,80	9,15	6,92	8,58	6,64	**8,08**	0,89
2.75	8,64	9,01	7,63	8,26	7,81	9,08	6,88	8,58	6,66	**8,06**	0,88
3.00	8,57	8,90	7,55	8,18	7,76	8,95	6,67	8,43	6,56	**7,95**	0,89

C

Values expressed relative to first time point set to 100 % (orange field of the upper table).

time (h)	pMBMECs, no treatment (Exp A) 1	2	3	mean	STDEV	pMBMECs, no treatment (Exp B) 1	2	3	mean	STDEV
start (0)	85,9	108,3	105,8	**100,0**	12,3	102,8	89,5	107,7	**100,0**	9,4
0.25	83,5	105,6	103,3	**97,4**	12,1	100,0	88,4	106,3	**98,2**	9,1
0.50	81,9	104,7	101,5	**96,0**	12,4	98,4	87,5	105,6	**97,2**	9,1
0.75	79,7	101,7	98,5	**93,3**	11,9	97,8	87,6	105,4	**96,9**	8,9
1.00	78,5	99,9	96,7	**91,7**	11,6	98,4	87,9	105,9	**97,4**	9,1
1.25	76,5	97,6	94,0	**89,4**	11,3	98,1	87,4	105,3	**96,9**	9,0
1.50	75,4	95,6	91,5	**87,5**	10,7	97,9	86,9	105,0	**96,6**	9,1
1.75	73,6	93,6	89,4	**85,5**	10,5	97,6	86,6	104,3	**96,2**	8,9
2.00	73,0	92,7	88,6	**84,7**	10,4	97,1	85,8	103,9	**95,6**	9,2
2.25	73,2	92,6	89,2	**85,0**	10,4	96,3	85,5	103,9	**95,2**	9,2
2.50	73,7	92,9	90,2	**85,6**	10,4	96,0	85,2	100,9	**94,0**	8,1
2.75	74,7	93,6	91,3	**86,6**	10,3	95,4	84,3	98,4	**92,7**	7,4
3.00	75,4	93,8	91,2	**86,8**	10,0	94,8	83,7	101,4	**93,3**	9,0

time (h)	pMBMECs +B78chOVA (Exp A) 1	2	3	4	5	6	7	8	9	mean	STDEV
start (0)	120,3	111,9	101,7	100,4	102,0	96,3	96,2	96,3	74,9	**100,0**	12,4
0.25	109,6	100,1	90,3	87,4	90,8	85,3	85,4	85,2	65,1	**88,8**	12,1
0.50	103,4	93,7	84,1	80,7	83,9	79,0	79,3	79,3	58,8	**82,5**	12,1
0.75	100,2	90,0	81,2	76,8	79,8	75,3	76,0	76,0	55,2	**78,9**	12,1
1.00	98,5	88,2	80,2	73,9	78,0	73,6	74,1	73,8	53,0	**77,0**	12,3
1.25	95,1	85,6	77,8	70,2	74,9	71,5	70,2	71,2	49,4	**74,0**	12,5
1.50	91,4	81,9	73,9	66,3	72,2	68,1	67,2	69,0	46,6	**70,7**	12,2
1.75	86,8	77,7	68,8	60,8	67,0	64,2	63,0	64,7	42,1	**66,1**	12,2
2.00	83,4	72,5	63,1	56,6	62,0	59,6	58,8	60,0	38,2	**61,6**	12,2
2.25	79,0	68,0	58,5	51,6	57,1	55,3	54,9	54,7	34,5	**57,1**	12,0
2.50	75,2	63,6	55,0	48,0	54,0	51,4	51,9	51,1	31,6	**53,5**	11,7
2.75	73,0	61,6	53,7	46,0	52,3	49,7	50,3	49,5	29,8	**51,8**	11,6
3.00	71,3	60,0	53,3	44,0	51,1	48,7	49,6	48,2	28,8	**50,6**	11,5

time (h)	pMBMECs +B78chOVA +GM6001 (20µM) 1	2	3	4	5	6	7	8	9	mean	STDEV
start (0)	**101,5**	**107,8**	**98,5**	**101,9**	**96,1**	**110,3**	**89,4**	**105,0**	**89,5**	**100,0**	7,4
0.25	**99,0**	**104,4**	**95,4**	**98,0**	**93,2**	**106,8**	**86,7**	**101,1**	**86,3**	**96,8**	7,2
0.50	**97,2**	**101,5**	**93,9**	**95,7**	**91,0**	**104,7**	**85,1**	**99,1**	**84,7**	**94,8**	6,9
0.75	**94,0**	**98,1**	**90,9**	**92,4**	**88,1**	**101,8**	**82,4**	**95,8**	**81,4**	**91,7**	6,8
1.00	**91,1**	**95,4**	**87,9**	**89,4**	**85,3**	**98,8**	**79,1**	**92,9**	**77,7**	**88,6**	7,0
1.25	**87,4**	**91,8**	**84,0**	**85,9**	**81,4**	**94,5**	**75,8**	**89,2**	**73,8**	**84,9**	7,0
1.50	**84,1**	**88,8**	**80,5**	**82,6**	**78,2**	**91,3**	**72,0**	**85,9**	**70,3**	**81,5**	7,1
1.75	**79,9**	**84,5**	**75,4**	**78,3**	**73,8**	**86,4**	**67,8**	**81,6**	**65,8**	**77,1**	7,1
2.00	**77,8**	**82,2**	**72,1**	**75,3**	**71,3**	**83,8**	**64,2**	**78,2**	**62,4**	**74,2**	7,4
2.25	**76,4**	**80,1**	**69,3**	**73,5**	**69,5**	**81,5**	**62,2**	**76,0**	**60,0**	**72,1**	7,5
2.50	**75,0**	**79,1**	**67,1**	**72,0**	**68,1**	**79,9**	**60,4**	**74,9**	**58,0**	**70,5**	7,7
2.75	**75,5**	**78,7**	**66,6**	**72,1**	**68,2**	**79,3**	**60,1**	**74,9**	**58,2**	**70,4**	7,7
3.00	**74,8**	**77,7**	**65,9**	**71,4**	**67,7**	**78,1**	**58,2**	**73,6**	**57,2**	**69,4**	7,8

Supplementary Table S2. **A** lists the initial absolute TEER values. **B** lists the absolute TEER values subtracted by mean TEER values of the empty filters. **C** shows the calculation of the data shown in Supplementary Figure 1B.
